# Expression-Guided In Silico Evaluation of Candidate *Cis* Regulatory Codes for *Drosophila* Muscle Founder Cells

**DOI:** 10.1371/journal.pcbi.0020053

**Published:** 2006-05-26

**Authors:** Anthony A Philippakis, Brian W Busser, Stephen S Gisselbrecht, Fangxue Sherry He, Beatriz Estrada, Alan M Michelson, Martha L Bulyk

**Affiliations:** 1 Division of Genetics, Department of Medicine, Brigham and Women's Hospital and Harvard Medical School, Boston, Massachusetts, United States of America; 2 Harvard/MIT Division of Health Sciences and Technology (HST), Harvard Medical School, Boston, Massachusetts, United States of America; 3 Harvard University Graduate Biophysics Program, Harvard Medical School, Boston, Massachusetts, United States of America; 4 Department of Pathology, Brigham and Women's Hospital and Harvard Medical School, Boston, Massachusetts, United States of America; University of California Berkeley, United States of America

## Abstract

While combinatorial models of transcriptional regulation can be inferred for metazoan systems from a priori biological knowledge, validation requires extensive and time-consuming experimental work. Thus, there is a need for computational methods that can evaluate hypothesized *cis* regulatory codes before the difficult task of experimental verification is undertaken. We have developed a novel computational framework (termed “CodeFinder”) that integrates transcription factor binding site and gene expression information to evaluate whether a hypothesized transcriptional regulatory model (TRM; i.e., a set of co-regulating transcription factors) is likely to target a given set of co-expressed genes. Our basic approach is to simultaneously predict *cis* regulatory modules (CRMs) associated with a given gene set and quantify the enrichment for combinatorial subsets of transcription factor binding site motifs comprising the hypothesized TRM within these predicted CRMs. As a model system, we have examined a TRM experimentally demonstrated to drive the expression of two genes in a sub-population of cells in the developing *Drosophila* mesoderm, the somatic muscle founder cells. This TRM was previously hypothesized to be a general mode of regulation for genes expressed in this cell population. In contrast, the present analyses suggest that a modified form of this *cis* regulatory code applies to only a subset of founder cell genes, those whose gene expression responds to specific genetic perturbations in a similar manner to the gene on which the original model was based. We have confirmed this hypothesis by experimentally discovering six (out of 12 tested) new CRMs driving expression in the embryonic mesoderm, four of which drive expression in founder cells.

## Synopsis

Although genome sequences and much gene expression data are readily available, the determination of sets of transcription factors regulating particular gene expression patterns remains a problem of fundamental importance. Tissue-specific gene expression in developing animals is regulated through the combinatorial interactions of transcription factors with DNA regulatory elements termed *cis* regulatory modules (CRMs). Although genetic and biochemical experiments allow the identification of transcription factors and CRMs, those experiments are laborious and time-consuming. Philippakis et al. introduce a new approach (termed “CodeFinder”) for quantifying the enrichment for particular combinations of transcription factor binding site motifs within predicted CRMs associated with a given gene set of interest, identified from gene expression data. The authors' analyses allowed them to discover a specific combination of transcription factor binding site motifs that constitute a core *cis* regulatory code for expression of a particular subset of genes in muscle founder cells, an embryonic cell population in the developing fruit fly *(Drosophila melanogaster)* mesoderm, and also led them to the discovery and subsequent experimental validation of novel, tissue-specific CRMs. Importantly, the CodeFinder approach is generally applicable, and thus could be used to support, refute, or refine a known or hypothesized *cis* regulatory code for any biological system or genome of interest.

## Introduction

A central challenge to determining the structure of genetic regulatory networks is the development of systematic methods for assessing whether a set of transcription factors (TFs) co-regulates a given set of co-expressed genes. Although classical genetics approaches allow the identification of key regulating TFs and the determination of their approximate ordering within the genetic hierarchy, demonstrating that a collection of TFs forms a combinatorial code acting to directly drive gene expression has required laborious experimental identification and perturbation of numerous individual *cis* regulatory modules (CRMs; [1]). To speed this process, several groups have recently demonstrated that computational approaches can rapidly identify CRMs with considerable accuracy [2–17], especially when performing computational searches with a collection of TFs known a priori to co-regulate. This is perhaps best exemplified by the dramatic progress made by several groups in discovering CRMs for genes expressed during segmentation of the Drosophila melanogaster embryo [2,3,6,14], a system where years of genetic screens have identified the regulating TFs [18]. In most biological systems, however, such a set of co-regulating TFs is either merely hypothesized or entirely unknown. Therefore, in order for these in silico approaches to effectively identify the *cis* component of regulation in novel biological systems (i.e., discover CRMs), additional computational methods are needed that can identify the *trans* component of regulation (i.e., the set of co-regulating TFs).

**Figure 1:**
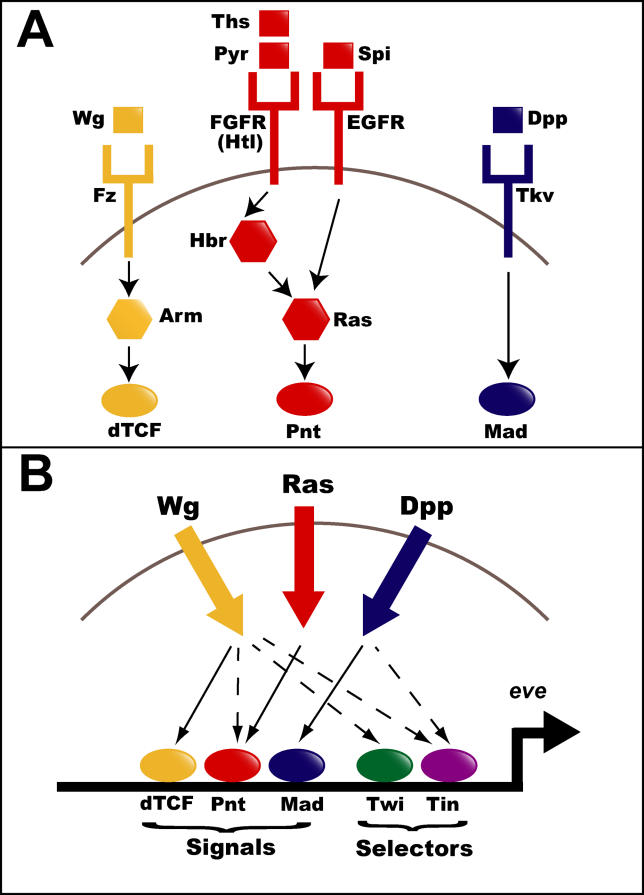
Genetic Regulation of Founder Cell Fates (A) Schematic of Wg, Ras, and Dpp signal transduction cascades responsible for specifying FC fates. Transmembrane receptors (fork-shapes), their ligands (squares), intracellular signaling molecules (octagons), and target TFs (ovals) are shown and colored by pathway. (B) Schematic of *eve* transcriptional regulation. Shown in thick solid arrows are the signaling inputs from the Wg, Dpp, and Ras pathways. Shown in thinner arrows are the genetic interactions linking these signals to their downstream TFs; solid arrows indicate interactions between proteins of the same pathway, and dotted arrows indicate known interactions between pathways. Colored circles indicate the five TFs (dTCF, Mad, Pnt, Twi, and Tin) known to drive *eve* expression within FCs.

To address this question in metazoan systems, we have developed an initial statistical framework for evaluating hypothesized transcriptional regulatory models (TRMs; i.e., sets of TFs that together co-regulate a target gene set through their combinatorial interactions at CRMs). As a model system, we have examined the regulation of a class of *Drosophila* myoblast genes for which a regulatory model has been previously hypothesized [19,20] and for which extensive transcriptional profiling datasets have been generated [21]. Muscle founder cells (FCs) are a sub-population of mononucleate myoblasts that are specified by the Wingless (Wg), Decapentaplegic (Dpp), and Ras signal transduction cascades acting in combination within the somatic mesoderm [22,23] (these pathways and some of their key regulators are schematized in Figure 1). Prior experimental work using the gene *even-skipped (eve)* to mark a single FC in each embryonic hemisegment provided a detailed model for the integration of these three signaling pathways at the transcriptional level: the TFs activated by the Wg, Dpp, and Ras pathways—T cell factor (dTCF), Mothers against dpp (Mad), and Pointed (Pnt), respectively—were demonstrated to bind a transcriptional enhancer driving expression of *eve* within dorsal FCs [19,20,24,25]. Additional tissue specificity was shown to be provided by two mesodermal selector TFs, Twist (Twi) and Tinman (Tin; Figure 1B). Thus, from this single enhancer, a combinatorial model of transcriptional regulation for genes expressed in FCs (especially those with expression in the dorsal mesoderm) was hypothesized, where exogenous signaling cues and endogenous tissue-specific TFs jointly establish the appropriate expression domain.

Guided by this genetic analysis of *eve* expression, a series of gene expression profiles has been determined for purified embryonic myoblasts by Estrada et al. [21]. In addition to profiling wild-type cells, these investigators performed expression array analyses of myoblasts in which the Wg, Dpp, Ras, and Notch pathways were variably perturbed by 12 informative gain-of-function (gof) and loss-of-function (lof) genetic manipulations (we note that the Notch pathway also functions in the mesoderm to distinguish the cell fates of FCs from those of fusion competent myoblasts [26]). Each of these 12 genetic perturbations was predicted, based on the example of *eve,* to increase or decrease expression of those genes with localized expression in FCs (henceforth referred to as “FC genes”). These 12 expression arrays were then combined into a single weighted ranking (henceforth referred to as the “composite FC ordering”), which was used to predict additional FC genes. Estrada et al. [21] performed over 200 in situ hybridizations on predicted FC genes from the top of this composite FC ordering, and their experiments (as well as a review of the literature) yielded a list of 159 validated FC genes.

In the present work, we utilize the expression data of Estrada et al. [21] to evaluate the roles of dTCF/Mad/Pnt/Twi/Tin as generalized regulators of FC gene expression. A previous computational scan for windows of sequence containing these five TFs successfully identified an additional enhancer for the gene *heartbroken (hbr)* that drove expression in dorsal FCs and contained matches to these five transcription factor binding site (TFBS) motifs, demonstrating that the example of *eve* was not unique [20]. However, the generality of the model could not be established by those two examples alone, and we therefore developed a method of quantifying enrichment for these five TFBS motifs in localized windows of non-coding sequences flanking or intronic to FC genes. Importantly, this approach, which we term “CodeFinder,” quantifies the relevance of not only each TF individually, but also of all combinations of the given set of TFs. From this analysis, we hypothesized that the *eve* TRM is unlikely to apply to all FC genes. Rather, we found that three TFs—Pnt, Twi, and Tin—are likely to regulate a specific subset of FC genes that share characteristic changes in their gene expression profiles in response to the genetic perturbations used by Estrada et al. [21]. Thus, by combining TFBS and gene expression data, our analysis allows a refinement of the initial model such that a subset of the original TFs appears to regulate a subset of FC genes. As a test of this hypothesis, we have empirically validated four candidate FC enhancers that conform to our modified TRM (as well as two additional enhancers driving expression in other domains of the embryonic mesoderm).

## Results

### FC Genes Are Enriched for Clusters of dTCF/Mad/Pnt/Twi/Tin Motifs in Their Flanking and Intronic Non-Coding Sequences

We first compiled from the literature, experimentally verified binding sites for each of the five TFs dTCF/Mad/Pnt/Twist/Tin (see Protocol S1). Additionally, we obtained a collection of 159 genes validated by in situ hybridization to be FC genes [21]; see Protocol S1). A common approach for determining whether a set of genes is targeted by a collection of TFs is to look for instances of the corresponding TFBS motifs immediately upstream of transcriptional start [27–29]. In preliminary analyses, we determined that the proximal 1–2 kb of flanking sequences upstream of these 159 FC genes were not significantly enriched for the dTCF/Mad/Pnt/Twist/Tin motifs relative to the corresponding regions of randomly selected genes taken as a background set (*p* > 0.05 after Bonferroni correction for multiple hypothesis testing, see Materials and Methods). Because we did not uncover any clues to the mechanisms underlying transcriptional regulation of FC genes from an analysis of their proximal promoter regions, we sought to develop a framework that could evaluate the over-representation of the dTCF/Mad/Pnt/Twist/Tin motifs in the extended flanking and intronic sequences of these genes.

In approaching this problem, we were influenced by the strategy of Mootha et al. [30], who looked at the aggregate behavior of entire gene sets, rather than individual genes, in analyzing gene expression microarray data. In their method, genes were ranked by expression change, and independently defined gene sets were then inspected to see if their positions within this ranking were non-randomly distributed. From that analysis, they were able to observe trends in the aggregate behavior of the gene set that were not significant when looking on a gene-by-gene basis. Our approach borrows from this method, but utilizes a sequence-based (rather than expression-based) method of ranking genes. Here, genes are ordered according to their enrichment for various combinations of TFBS motifs in localized windows of sequence, and a given foreground gene set is then inspected to see if its distribution of ranks within this list is non-random. The goal of this analysis is to uncover effects that are otherwise small in size, but that can be statistically quantified.

**Figure 2:**
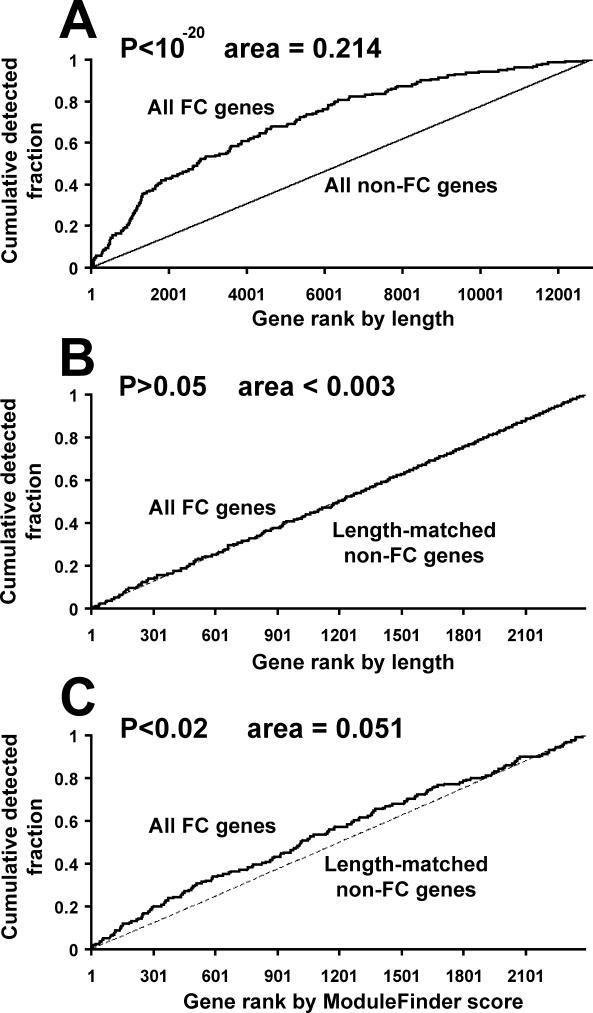
Inspection of an FC TRM Composed of dTCF/Mad/Pnt/Twi/Tin (A) Detection rate of the 159 known FC genes as compared to all other D. melanogaster genes, when genes are ranked by the amount of associated non-coding, non-repetitive sequence. The X-axis indicates a given cutoff rank; the Y-axis indicates the fraction of either the 159 FC genes (solid line) or the non-FC genes (dotted line) observed to have a length greater than the corresponding cutoff rank. (B) Detection rates of the 159 known FC genes (solid line) and a set of length-matched background sequences (dashed line; see Materials and Methods) when ranked by length; it can be seen that these curves are largely overlapping. (C) Detection rates of the 159 known FC genes as compared to length-matched background sequences, when genes are ranked by ModuleFinder scores using a scan in which any combination of the five TFs can contribute to the score. Again, the X-axis indicates a given cutoff rank and the Y-axis indicates the fraction of the 159 FC genes (solid curve) or background sequences (dotted curve) with ModuleFinder scores better than the given cutoff rank. For all panels, the area between these curves is computed, and its statistical significance is computed using the WMW-statistic (see Materials and Methods).

For each of the 159 validated FC genes, we searched the entire non-coding upstream, downstream and intronic regions with a CRM identification tool named ModuleFinder that was previously developed by our group [31]. This program is one of a number of approaches that scores windows of genomic sequence according to the degree of TFBS clustering and/or evolutionary conservation [3,7,9,13,16,31–33] (our approach most resembles that of Lifanov et al. [13], but extends it by incorporating a measure of evolutionary conservation in addition to binding site clustering). Next, we assigned to each gene the ModuleFinder score of the most significant window adjacent to it (i.e., a ModuleFinder “hit”; see Materials and Methods). Under the hypothesis that dTCF/Mad/Pnt/Twi/Tin are widely acting regulators of FC genes, we anticipated that many of these 159 genes would be enriched for significant ModuleFinder scores in their surrounding non-coding sequence, as compared with a suitable background set (Figure 2).

In an initial application, we used the remaining D. melanogaster genes as a background set. As this was being implemented, however, another group observed that regulatory genes such as TFs and kinases typically had more flanking sequence [34]. Our FC gene list was substantially enriched for TFs and signaling proteins [21]; consistent with this, we observed that the median amount of non-coding, non-repetitive sequence flanking these 159 FC genes was approximately 15.2 kb, whereas the remaining D. melanogaster genes had a median of only approximately 3.4 kb of non-coding, non-repetitive flanking sequence (*p* < 1 × 10^−20^ by Wilcoxon-Mann-Whitney [WMW] statistic, Figure 2A). In order to remove the possibility that any observed enrichment for ModuleFinder hits could be explained solely by a larger search space, we selected a length-matched set of D. melanogaster background sequences. We note that this matching was performed such that not only the average or median lengths between foreground and background regions were matched, but rather so that the entire foreground and background length distributions were matched (see Figure 2B and Materials and Methods).

In order to evaluate the enrichment of the TFBS motifs under consideration in the foreground gene set, we sought a metric that could quantify the degree to which the foreground genes ranked higher than the background genes on the basis of their ModuleFinder scores. The WMW statistic specifically tests this null hypothesis [35]; however, the *p*-value attached to it reflects both the degree of foreground enrichment and the sample sizes of the foreground and background. Therefore, we used an additional measure of enrichment that is less dependent on the number of foreground and background genes, so that effects between gene sets of different sizes could be compared. For this we utilized the area between the detection rate curves shown in Figure 2, because one can show that the area between these curves is a geometric representation of the WMW statistic scaled to be independent of sample size (see Materials and Methods).

Using criteria in which any combination of the five TFs of interest can contribute to the ModuleFinder score for each gene, we observed enrichment for high-scoring ModuleFinder hits adjacent to FC genes as compared to the length-matched background sequences (*p* < 0.02 by WMW statistic, area = 0.051; see Figure 2C). The degree of this enrichment was slight, however, suggesting one of the following two scenarios: 1) the five TFs (dTCF/Mad/Pnt/Twi/Tin) are targeting a large fraction of the 159 FC genes, but our basic approach of quantifying binding site enrichment has limited statistical power to observe it, or 2) only a subset of the 159 FC genes is targeted by some combination of these five TFs with higher frequency than the genomic background. In order to address this second possibility, we set out to utilize the gene expression data of Estrada et al. [21] to systematically identify whether particular subsets of the original 159 FC genes are likely to be targeted by the hypothesized FC TRM or a modified version of it.

### Differential Response of FC Genes to Gof of Pnt, a Ras-Dependent TF

**Figure 3:**
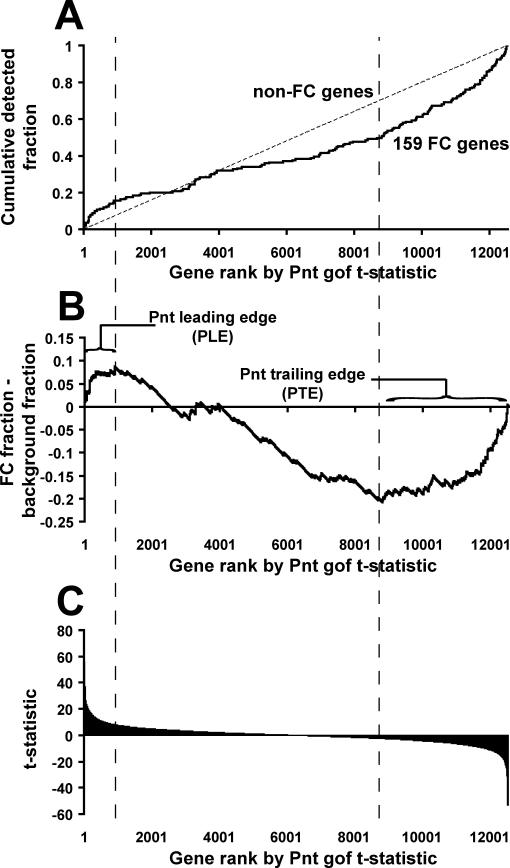
Changes in Expression of FC Genes in a Pnt gof Mutant Background (A) Detection rate of the 159 known FC genes in a Pnt gof expression profile. All genes are ranked according to the t-statistic (see Estrada et al. [21]) indicating their up- or down-regulation in a Pnt gof mutant background (the most up-regulated genes are positioned at the left). As in Figure 2, detection rates of the 159 known FC genes (solid line) and all other genes (dashed line) are shown. (B) Difference between the detection rate curves of (A); leading and trailing edges indicate the points of maximal difference. (C) t-statistics for all genes in the Pnt gof expression profile.

In constructing the composite ordering of newly identified FC genes, Estrada et al. [21] weighted each of the 12 mutant expression profiles according to the degree to which a training set of 33 previously known and validated FC genes responded in the expected fashion (i.e, were up- or down-regulated) within that genetic background. A somewhat surprising result of their analysis was that, although arrays performed on gof genetic backgrounds corresponding to upstream regulators of the Ras pathway (i.e., constitutively activated forms of *EGFR, FGFR, Ras,* and *armadillo+Ras*) caused the training set of FC genes to be up-regulated, gof of Pnt—a TF acting downstream of these factors—had little aggregate effect on the same FC gene training set [21] (see Figure 3).

Since the model enhancer *eve* responded strongly to Pnt gof and was known to contain functionally validated TFBSs for Pnt [19], we re-inspected the Pnt expression profile to see how all FC genes responded to Pnt gof. We first ranked all genes according to their up- and down-regulation in the Pnt gof background, and we then looked at the positions of the 159 FC genes within this ranking (see Figure 3A). Interestingly, the curve showing the rate at which these 159 FC genes were detected had a sigmoidal shape, suggesting that Pnt gof has a dual role as both an activator and a repressor of different subsets of FC genes. Because the Pnt gof profile was weighted so little in constructing the composite ordering of FC genes, it is important to note that the shape of this curve is not a result of ascertainment bias in how FC genes were discovered. In addition, since this microarray experiment involved a strong, constitutively activated form of Pnt [19,21], it is inferred that the observed repressive effect is likely to be indirect.

Utilizing the “leading edge” analysis of Subramanian et al. [36], we took as a foreground gene set those genes ranking higher than the point at which the foreground and background detection curves maximally diverged (Figure 3B). We shall henceforth refer to this gene set as the “Pnt leading edge” (PLE). This set of 25 genes corresponded roughly to those genes up-regulated in the Pnt gof array with a q-value of 0.1 or less, or equivalently as having a t-statistic score of roughly 7.9 or greater (Figure 3C; see Estrada et al. [21] for details relating to statistical analyses of microarrays). Thus, in calling these genes up-regulated in the Pnt gof background, one would expect only a few to be false positives. As further confirmation that the PLE was not merely a statistical artifact, we independently validated the microarray results by performing in situ hybridizations in a Pnt gof background for 15 of the 25 genes in the PLE, and observed that 14 of them did, in fact, have visible expansion of their embryonic expression domains.

**Figure 4:**
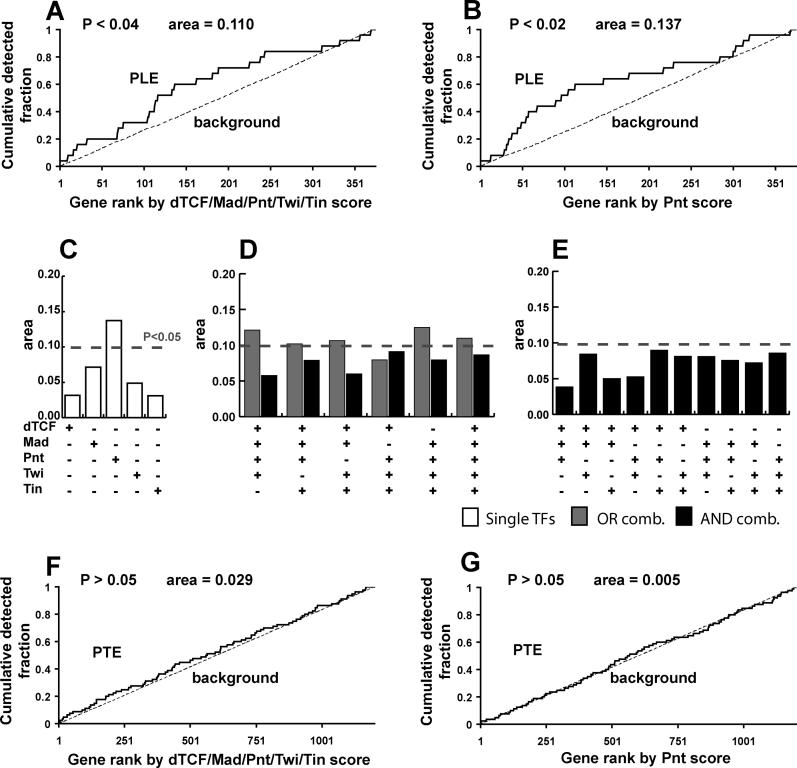
Enrichment for the FC TRM in PLE Genes (A) PLE and background genes were scanned by ModuleFinder using dTCF/Mad/Pnt/Twi/Tin and sorted by score in decreasing order. As in Figure 2, detection curves for PLE genes and non-PLE genes are shown. (B) PLE and background genes were scanned by ModuleFinder using only the Pnt motif and sorted in decreasing order. (C–E) Area between PLE and non-PLE detection curves is shown when scanning with the TFs dTCF/Mad/Pnt/Twi/Tin either individually (C), with all AND and OR combinations involving four or five TFs (D), or all AND combinations involving three TFs (E). (F–G) Dotted lines indicate threshold statistical significance values of *p* < 0.05, as computed by WMW. Also shown are the detection rate curves using the PTE as a foreground set using the OR combination dTCF/Mad/Pnt/Twi/Tin (F), as well as the Pnt motif alone (G).

When we inspected the PLE (Figure 4), we noticed that it showed greater enrichment for the five TFs dTCF/Mad/Pnt/Twi/Tin (area = 0.110; *p* < 0.04; Figure 4A) than the original collection of 159 FC genes (area = 0.051; *p* < 0.02). Because this gene set was defined to be the collection of FC genes most up-regulated in a Pnt gof background, we inspected whether the Pnt TFBS motif was, by itself, enriched in the non-coding sequences associated with these genes. Surprisingly, we observed that it was more enriched than the pooled collection of all five TFs (area = 0.137; *p* < 0.02; Figure 4B), suggesting that one or more of the TFs under consideration was not contributing to the observed foreground enrichment. Therefore, we developed a systematic means of determining which TFBS motifs and combinations of TFBS motifs were most likely contributory, an approach which we call CodeFinder.

### CodeFinder Provides a Systematic Examination of TFBS Combinatorics in Reference to a Set of Co-Expressed Genes

Given foreground and background gene sets *F* and *B*, and a set of transcription factor binding site motifs *M*, it is desired to provide confirming or refuting evidence for the over-representation of *M* in *F* relative to *B*. Three concerns must be addressed in order to effectively evaluate combinatorial interactions between the TFs considered. First, any given motif set *M* is unlikely to be necessary or sufficient for regulation of *F* (i.e., not all genes in *F* will actually be targeted by *M,* and there may be motifs other than those of *M* that contribute to the regulation of *F*). Thus, the metric must be able to quantify even only slight degrees of foreground enrichment. Second, because the score responsible for ranking the foreground and background genes is a linear sum of scores for the input motifs, if one of the input motifs is not enriched in *F,* then omitting it from the search should result in a greater degree of left-shifting for *F,* as it is acting only to increase score variability (noise) in *F* and *B*. Hence, it is necessary to inspect subsets of TFBS motifs comprising the TRM. Finally, there is the possibility that a combination of TFBS motifs shows increased enrichment relative to its subsets not because the combination is truly co-regulating, but because the genes in *F* are being targeted by overlapping subsets of that combination. Hence, a mechanism is needed to distinguish between these possibilities.

To address these concerns, we systematically inspected combinations of the five TFs dTCF/Mad/Pnt/Twi/Tin. First, we looked at each of the five TFs individually and observed that only Pnt was significantly enriched in the foreground set (areas reported in Figure 4C). Next, we inspected whether genes up-regulated in the Pnt gof background were likely to follow the *eve* TRM by simultaneously having binding sites for all (or nearly all) five of the TFs dTCF/Mad/Pnt/Twi/Tin. To address this, we looked at all combinations involving four or five TFs and added the constraint that any window not containing at least one match to each of the motifs utilized in the search be scored as “0” (we henceforth refer to these as “AND” combinations of the motifs, and refer to those combinations where this restriction is not imposed as “OR” combinations; thus the curve shown in Figure 2C is the OR combination involving all five TFs). In looking at AND and OR combinations involving four or five of the TFs, we observed that the AND combinations reliably showed less foreground enrichment than their OR counterparts (Figure 4D). Finally, we looked at all AND combinations involving three combinations of the TFBS motifs in order to inspect putative modes of combinatorial regulation between the preceding two extremes, and observed that nearly all combinations showed only slight foreground enrichment. Indeed, in looking at all combinations of the five TFBS motifs, we observed that none showed as much foreground enrichment as Pnt alone (see Figure 4C–4E and Table S1).

Throughout this analysis, the foreground and background sequence sets remained invariant, but their overall ordering changed with each combination of motifs. Thus, it is important to note that combinations involving different numbers of motifs can be compared on equal footing, since a smaller number of relevant motifs can cause the foreground sequences to rank more highly than a larger set involving irrelevant motifs. From this analysis, we conclude that: 1) the TFBS motif Pnt is likely to directly target a substantial fraction of genes in the PLE, and 2) that although the other four factors may each be working with Pnt to participate in the regulation of some genes in this set, it is unlikely that there is a single combination responsible for targeting all of these genes.

Finally, one of the genes in the PLE is Yan, an Ets-domain transcriptional repressor [37] (recall that Pnt is also an Ets domain protein, but is known to act as a transcriptional activator [38]). We inspected the possibility that Yan might be responsible for the down-regulation of the Pnt trailing edge (PTE) gene set, possibly in combination with some of the other TFs under consideration. Here, when we looked at the OR combination involving all five TFBS motifs, we saw no statistically significant enrichment (Figure 4F). Similarly, when looking at each TFBS motif individually, we did not see especially strong enrichment for any of the motifs, including Pnt (i.e., the Ets motif which should be similar to the Yan motif; Figure 4G); thus, we can provide no evidence that Yan is acting to directly regulate this gene set. (All Boolean combinations for the Pnt leading and trailing edges are given in Table S1.)

### An Expression Cluster of FC Genes Enriched for Pnt AND Twi AND Tin

**Figure 5:**
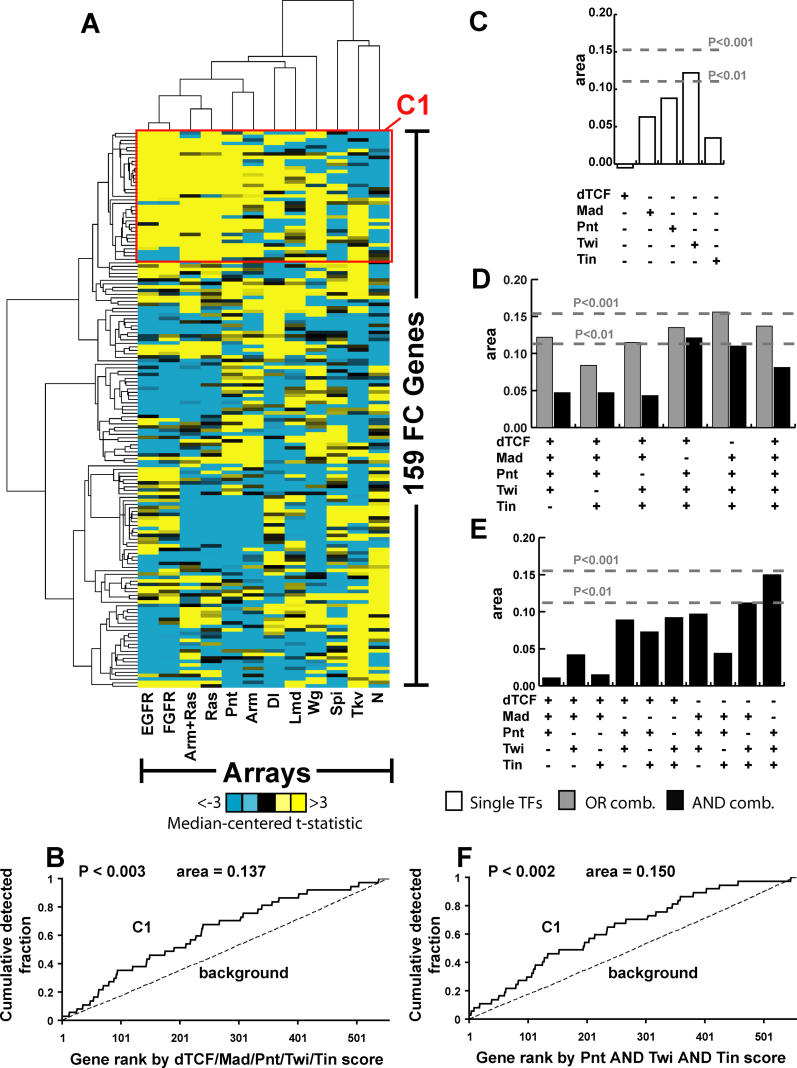
An Expression Cluster of Genes Enriched for Pnt AND Twi AND Tin (A) Clustering of the 159 FC genes and the 12 expression profiles of Estrada et al. [21], using self-organizing map clustering followed by hierarchical clustering. Note that all columns are median-centered. The red box indicates a gene cluster (C1) that contains *eve* and whose genes show similar expression profiles. Here, abbreviations are EGFR = *EGF receptor* gof; FGFR = *FGF receptor* gof; Arm+Ras = *armadillo* and *Ras* gof; Ras = *Ras* gof; Pnt = *pointed* gof; Arm = *armadillo* gof; Dl = *Delta* lof; Lmd = *Lameduck* lof; Wg = *wingless* lof; Spi = *spitz* lof; Tkv = *thickveins* gof; N = *Notch* gof. (B) Detection rate curves for the OR combination of dTCF/Mad/Pnt/Twi/Tin using C1 as a foreground gene set. (C–E) Area between C1 and non-C1 detection curves is shown when scanning with the TFs dTCF/Mad/Pnt/Twi/Tin either individually (C), with all AND and OR combinations involving four or five TFs (D), or all AND combinations involving three TFs (E). Dotted lines indicate threshold statistical significance values of *p* < 0.01 and *p* < 0.001, as computed by WMW. (F) Detection rate curves for Pnt AND Twi AND Tin combinations using C1 as a foreground gene set.

Having utilized the Pnt gof expression profile to identify a gene set likely to be directly targeted by Pnt, we wanted to see if we could utilize the entire collection of expression profiles from Estrada et al. to identify one or more additional gene sets enriched for the TFs comprising the hypothesized TRM (Figure 5). We performed self-organizing map [39] clustering followed by hierarchical clustering [40] on the 159 FC genes validated by in situ hybridization, clustering both profiles and genes (Figure 5A; see Materials and Methods). We note that, because so many of the 159 FC genes were up-regulated in the four genotypes in which proximal Ras pathway components were activated *(Ras, arm+Ras, FGFR, EGFR),* we first median-centered the columns (but not the rows) so that it was possible to visualize a response gradient in these conditions.

The FC gene set indicated as Cluster 1 (C1) contained *eve;* moreover, genes in C1 responded to the genetic perturbations of Estrada et al. [21] in a manner most similar to what had been observed in the original analysis of *eve* expression [19] (Figure 5A). We therefore determined if the aggregate collection of 37 genes found in C1 was enriched for some combination of motifs corresponding to the five TFs known to regulate the FC expression of *eve*. When we looked at the OR combination involving all five TFBS motifs, we again observed that it showed greater enrichment than did the original collection of 159 FC genes (Figure 5B). Therefore, we repeated our CodeFinder analysis in order to identify those combinations of TFBS motifs likely to be involved in the direct regulation of these genes. When we inspected each of the five TFBS motifs individually, we observed that Pnt and Twi each showed enrichment but, unlike the PLE genes, the degree of this enrichment was not as great as the OR combination involving all 5 TFs (Figure 5B and 5C). Next, we inspected all OR and AND combinations involving four or five TFBS motifs. Here, we also observed that AND combinations reliably showed less foreground enrichment than their OR counterparts (Figure 5D). Finally, when looking at all AND combinations involving three TFBS motifs, we observed that the combination Pnt AND Twi AND Tin showed especially strong enrichment (Figure 5E and 5F), suggesting that these three TFs might jointly target many of the genes in C1 (values for all combinations are given in Table S1).

### A Second Application of CodeFinder to Genes Expressed in Subsets of Cells in the Developing Fly Wing

**Figure 6:**
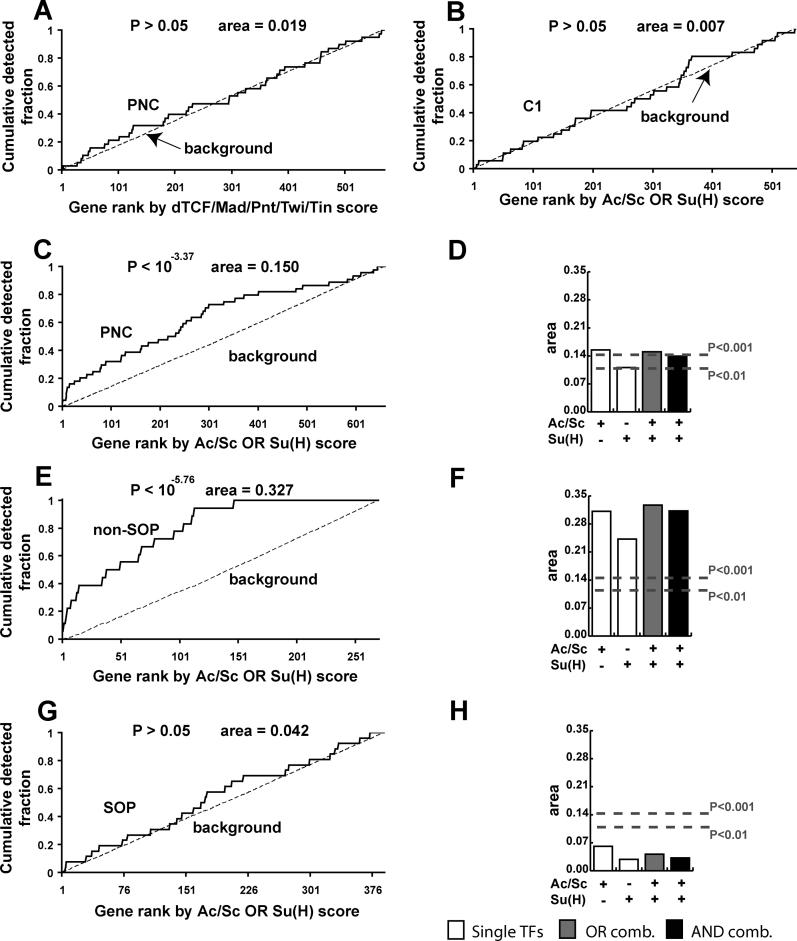
Analysis of PNC Genes and Their Associated TRM (A) Detection rates of PNC genes (after removing seven genes that are also FC genes) as compared to background regions using the OR combination of dTCF, Mad, Pnt, Twi, Tin (negative control). (B) Detection rates of C1 genes (after removing genes that are also PNC genes) as compared to background regions using the combination Ac/Sc OR Su(H) (negative control). (C) Detection rate of PNC genes as compared to non-PNC genes using Ac/Sc OR Su(H). (D) Area between PNC and background region detection rate curves for all AND and OR combinations of Ac/Sc and Su(H). (E) Detection rate of non-SOP genes as compared to background regions using Ac/Sc OR Su(H). (F) Area between non-SOP and background region detection rate curves for all AND and OR combinations of Ac/Sc and Su(H). (G) Detection rate of SOP genes as compared to background genes using Ac/Sc OR Su(H). (H) Area between SOP and background region detection rate curves for all AND and OR combinations of Ac/Sc and Su(H).

In order to test the generality of our approach, we used our CodeFinder framework to examine a second developmental system of comparable complexity and for which similar data were available (Figure 6). Reeves and Posakony recently characterized groups of genes that are expressed during development of the D. melanogaster peripheral nervous system [17]. This group performed expression profiling on purified proneural cluster cells (PNCs) of the larval wing, after which those genes determined to be up-regulated by the expression arrays were verified by in situ hybridization. This group hypothesized that the Achaete/Scute (Ac/Sc) motif, acting in conjunction with the Notch-dependent TF, Suppressor of Hairless, (Su(H); see below), formed part of a *cis* regulatory code driving the expression of many PNC genes.

From the supplementary data of Reeves and Posakony, we obtained a list of 44 genes validated by in situ hybridization to be expressed in PNCs [17]. As a computational negative control, we checked that this gene set was not enriched for targets of dTCF OR Mad OR Pnt OR Twi OR Tin (Figure 6A); similarly, we checked that the C1 gene list was not enriched for targets of Ac/Sc OR Su(H) (Figure 6B). In each case, there was no statistically significant enrichment (*p* > 0.05). For this analysis, there were seven genes in common between the FC and PNC gene lists, so these were removed from each set. Note that we also looked for enrichment of Ac/Sc OR Su(H) TFBS motifs in all 159 FC genes (see Figure S1) but observed faint enrichment (*p* < 0.03 by WMW; area = 0.049), which could reflect a slight biological role for these two TFs in regulating some FC genes. For example, one member of the *Ac*/*Sc* complex, *lethal of scute,* is expressed in the mesoderm and known to be involved in the regulation of FC fate [41]. Similarly Su(H), acting in the Notch pathway, is known to regulate the asymmetric cell division that establishes individual FC identities [42].

When we looked for enrichment of Ac/Sc and Su(H) motifs in the sequences surrounding PNC genes, we observed strong enrichment for these motifs, especially Ac/Sc (Figure 6C and 6D). Also, Reeves and Posakony subdivided the expression domains of these 44 PNC genes into two classes: one class composed of 26 genes expressed only in sensory organ precursors (SOPs; a subset of cells derived from the PNC that eventually become sensory neurons), and another class of 18 genes expressed in non-SOP cells (in some cases overlapping with SOPs). They further hypothesized that these two classes are under distinct regulatory programs, where activation of the Notch pathway promotes the non-SOP cell fate at the expense of the SOP cell fate; thus, non-SOP genes—such as those of the *enhancer of split* complex—should be enriched for targets of the Notch-activated TF Su(H), as well as for the proneural TFs, Ac/Sc [17,43], whereas SOP genes should be enriched for only Ac/Sc sites. In order to evaluate this hypothesis, we measured the degree of enrichment for the Ac/Sc and Su(H) motifs in each of these two classes individually. We observed greater enrichment for these motifs in the non-SOP class than the collection of all PNC genes (Figure 6C–6F). For the SOP class, we were unable to observe strong enrichment for either the Ac/Sc or the Su(H) motifs (Figure 6G and 6H). Here, it should be noted that there are individual genes in the SOP class such as *neuralized* that are very enriched for the Ac/Sc motifs. Nonetheless, our analysis suggests that perhaps not all SOP genes are direct targets of the Ac/Sc TFs, or at least that it is difficult to see this enrichment without also performing the computational searches with motifs for additional, co-regulating TFs.

### Validation of Novel FC CRMs Enriched for the Pnt, Twi, and Tin Motifs

**Figure 7:**
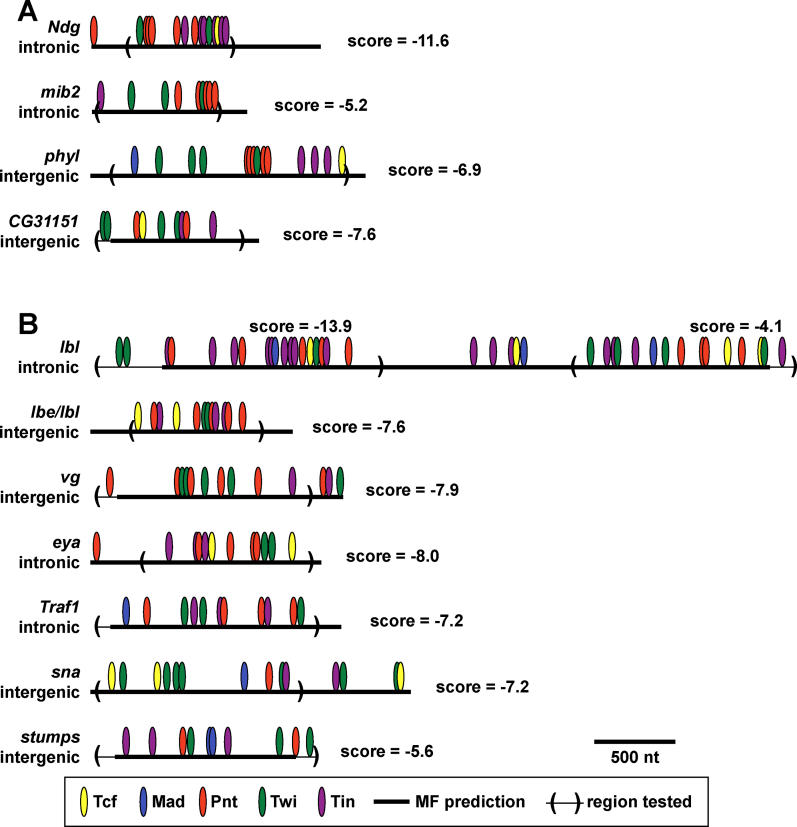
Schematic Representation of Tested Regions Associated with FC Genes The ModuleFinder prediction, TFBS composition, ModuleFinder score, genomic location and actual genomic region tested from regions associated with FC genes from C1 (A) or not included in C1 (B).

**Figure 8:**
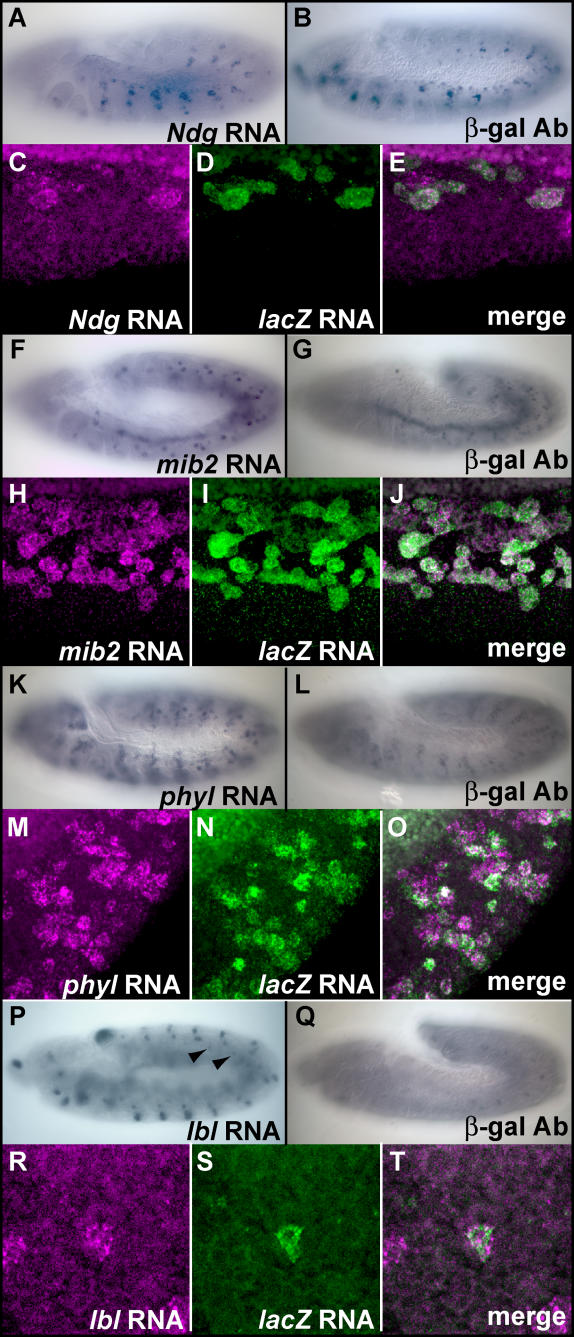
Empirical Validation of Predicted FC Transcriptional Enhancers Expression of *Ndg* (A), *mib2* (F), *phyl* (K), and *lbl* (P) mRNA in stage 11 wild type embryos detected by in situ hybridization. Arrowheads in (P) highlight *lbl*-expressing FCs. β-galactosidase expression from *Ndg*-*lacZ* (B), *mib2*-*lacZ* (G), *phyl*-*lacZ* (L), and *lbl*-*lacZ* (Q) constructs in stage-11 embryos detected by immunohistochemistry. Fluorescent in situ hybridization analysis of stage-11 embryos for *Ndg* (C), *lacZ* (D) mRNA, and merge (E) from *Ndg*-*lacZ* embryos; *mib2* (H), *lacZ* (I) mRNA, and merge (J) from *mib2*-*lacZ* embryos; *phyl* (M), *lacZ* (N) mRNA, and merge (O) from *phyl*-lacZ embryos; and *lbl* (R), *lacZ* (S) mRNA and merge (T) from *lbl*-lacZ embryos.

The preceding analyses suggested a TRM comprising Pnt AND Twi AND Tin targets many of the 37 FC genes found in C1. To test this hypothesis, we evaluated the in vivo functions of 12 candidate CRMs selected from the class of all ModuleFinder hits containing matches to the Pnt, Twi, and Tin TFBS motifs and located in the flanking or intronic sequences of the 159 FC genes (these 12 candidate CRMs are graphically depicted in Figure 7). Four of the candidate CRMs were associated with genes in C1 *(Nidogen [Ndg], mindbomb2 [mib2], phyllopod [phyl], CG31151),* and eight were associated with one of the 122 FC genes not found in C1. Note that Table S2 gives the genomic coordinates and number of dTCF/Mad/Pnt/Twi/Tin TFBS motif matches for the highest scoring ModuleFinder hit for every gene in C1 or the PLE, as well as detailed information on these 12 tested regions. As shown in Figure 8, four of these 12 candidate CRMs were found to direct *lacZ* expression in somatic muscle FCs that co-express with the endogenous gene *(Ndg, phyl, mib2, ladybird late [lbl]),* a result confirmed by double fluorescent in situ hybridization with *lacZ* and gene-specific probes; of note, three of the four genes associated with these enhancers were in C1 *(Ndg, phyl, mib2)*. (See Materials and Methods for experimental details)

The enhancer for *Ndg* (Figure 8A–8E) is found in the first intron of the gene and drives expression in *Ndg*-expressing FCs. This example is particularly interesting since *Ndg* encodes a basement membrane protein, whereas the two previously confirmed FC TRM target genes were a transcription factor *(eve)* and a component of the Ras signaling cascade *(hbr),* implying that the TFs under consideration are acting as direct transcriptional regulators at both proximal and distal nodes in the myogenic regulatory network. The enhancer for *mib2* (Figure 8F–8L), located in the third intron of the gene, drove expression in both somatic FCs and the visceral mesoderm (VM). The gene *mib2* is expressed in both domains, suggesting that either similar mechanisms regulate VM and FC gene expression or that two separable mesodermal enhancers are located in the tested region (the former possibility is consistent with the known roles of Twi, Tin, and Ras signaling in VM development [44,45]). The tested window for *phyl* is located downstream of the coding region and was found to drive expression in *phyl* positive FCs (Figure 8K–8O). The last of the tested ModuleFinder windows is located near a non-C1 FC gene, *lbl* (Figure 8P–8T*),* which encodes an NK homedomain transcription factor TF known to be expressed in numerous embryonic tissues, including one somatic muscle FC. The enhancer for *lbl* is located in the large first intron of the gene and directs expression in this single FC. In summary, three of four tested C1 enhancers, but only one of eight non-C1 enhancers, faithfully recapitulated expression of the associated FC gene.

Of the eight remaining tested FC CRM candidates, three (the regions labeled as *CG31151* in Figure 7A and *sna, lbl/lbe* in Figure 7B) failed to drive any detectable embryonic *lacZ* expression, and three (the regions labeled as *stumps, Traf1* and *eya* in Figure 7B) drove *lacZ* expression in a pattern that failed to co-express with the endogenous gene (unpublished data). Interestingly, the final two candidate FC CRMs drove *lacZ* expression in non-FC mesodermal cells identically to the endogenous gene. These included a region downstream of *vestigial* (labeled as *vg* in Figure 7B) that was active in the *vg*-expressing wing disc adepithelial cells (and also some epithelial cells; unpublished data), and another intronic region of *lbl* that was functional in *lbl*-expressing heart cells (Michaud et al., unpublished data).

## Discussion

We have described an easily implemented, controlled approach (termed “CodeFinder”) for evaluating the degree to which a hypothesized transcriptional regulatory code acts to drive the expression of an independently derived gene set. CodeFinder integrates TFBS and expression profiling information by examining the statistical overrepresentation of particular TFBSs (or combinations thereof) in the non-coding sequences of co-expressed genes. This approach provides the beginnings of a general framework that can be applied to higher metazoan genomes, as it considers not only the proximal promoter regions of genes, but also their extended upstream, downstream and intronic regions, while controlling for the difficulty that genes may not have homogeneous amounts of flanking sequence [34]. In addition, our approach does not require that the collection of TFs under consideration be genetically necessary or sufficient, because we allow for the possibilities that some of the hypothesized TFs are not directly contributory to the *cis*-regulatory code, or that there are unknown, additional TFs that are contributory. This is accomplished by developing a quantitative metric for the degree of enrichment for TFBS motifs among a foreground gene set. Importantly, this metric allows both different combinations of TFs and different gene sets to be compared on equal footing, so that an initially hypothesized TRM or foreground gene set can be refined.

We have applied CodeFinder to evaluate the roles of five TFs, dTCF/Mad/Pnt/Twi/Tin, as regulators of gene expression in muscle FCs. When inspecting an aggregate collection of 159 FC genes, we observed significant yet faint enrichment for these TFs. However, restricting to a subset of FC genes defined by up-regulation in a Pnt gof background amplified this enrichment; moreover, we observed that the bulk of this enrichment was due to the single TF Pnt (as might be expected in this genetic perturbation). Similarly, by restricting to a subset of genes defined by having common expression profiles across the genetic perturbations of Estrada et al. [21], we again observed that this foreground enrichment could be amplified. Here, however, we observed that most of the signal was due to the TFBS motif combination Pnt AND Twi AND Tin.

**Figure 9:**
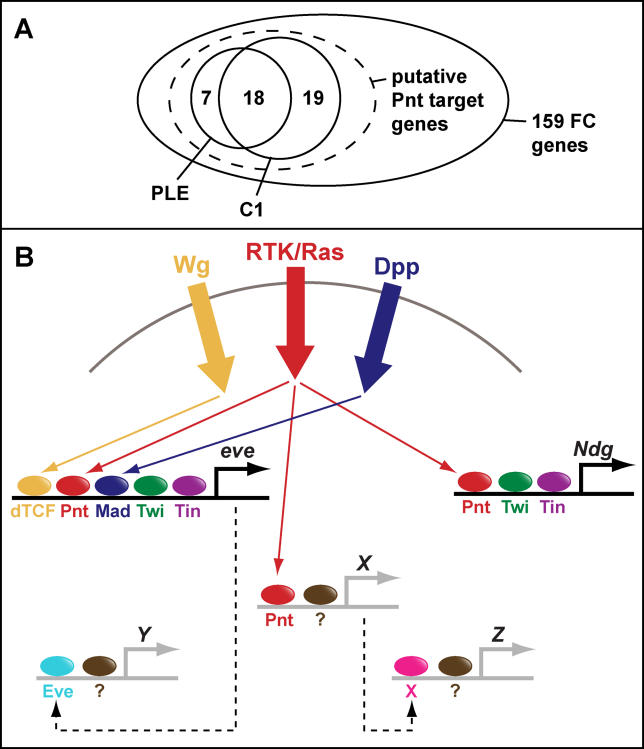
Summary of New Hypotheses Derived from the Present Analysis (A) Venn diagram depicting various FC gene subsets. Cluster 1 (C1) and the Pnt leading edge (PLE) genes are likely only a subset of all Pnt target genes (dashed ellipse), and additional FC genes appear to be unresponsive to Pnt. (B) Schematic of complexities in FC gene regulation. Analysis of the *eve* mesodermal enhancer initially directed our attention to the TFs dTCF, Pnt, Mad, Twi, and Tin. CodeFinder analysis and subsequent experimental validation implicated a subset of these TFs (Pnt, Twi, Tin) in the regulation of genes from C1, as exemplified by *Ndg*. Additional (non-C1) genes are predicted to respond to Pnt in combination with other factors yet to be determined (X; grey lines represent hypothetical enhancers). Still other classes of FC genes will respond to different codes, which may include input from FC genes known to encode TFs.

Interestingly, C1 and the PLE overlap at 18 genes (Figure 9). We observed that most of the genes in C1 were also at least somewhat up-regulated in the Pnt gof profile (30 out of the 37 genes showed expression change greater than 0, and only one gene was down-regulated in the Pnt gof profile with a *q*-value of 0.05). Thus, we hypothesize that there is a class of FC genes likely to be targeted by Pnt, of which the PLE is an especially high-confidence subset and C1 is another, overlapping subset (Figure 9A). Although this hypothesized class of genes would share Pnt as a direct regulator, it need not be commonly and exclusively regulated by any additional TFs (i.e., they need not all be targets of a single TRM). Thus, genes in C1 (e.g., *Ndg*) could be a subset of the Pnt target genes that are also targeted by Twi and Tin, whereas the other Pnt target genes might be regulated by one or more other TFs in addition to Pnt (Figure 9B). Indeed, a tissue- or cell type-specific selector other than Twi or Tin might be expected to act in conjunction with Pnt to confer specificity to the generic Ras signal that is mediated at the transcriptional level by Pnt [19]. Supporting this hypothesis 34/37 genes in C1 have at least one ModuleFinder hit scoring below −4.0 and containing Pnt AND Twi AND Tin, whereas 5 of 7 genes in the PLE, but not in C1 do *not* have such a corresponding hit. Additionally, it should be noted that even for C1, the combination of Pnt AND Twi AND Tin is unlikely to be genetically sufficient, as there is substantial overlap between the distributions of ModuleFinder scores between genes in C1 and background genes. Thus, further TRM complexity is expected to account for the heterogeneity of gene expression among individual FCs [21].

The enhancer discovered for an FC gene not in C1 *(lbl),* as well as the two enhancers discovered driving expression in other mesodermal cells (cardiac and adepithelial), highlight that there are likely to be additional gene sets targeted by some combination of dTCF/Mad/Pnt/Twi/Tin, most likely in addition to other, currently unknown, motifs. For the example of *lbl*, although it was not observed to be responsive to Pnt gof as determined by microarray t-statistics and fold-changes, we argue that the Ets-domain motifs in its FC enhancer may yet be meaningful as lof of *Yan* (an Ets domain transcriptional repressor) increases the number of *lbl*-positive FCs, suggesting that *Yan* normally represses the *lbl* FC enhancer in the absence of Ras/MAPK signaling [46]. For FC genes such as *lbl* that are not in C1 or the PLE, however, we do not observe enrichment for these TFs beyond the genomic background rate. This suggests that the appropriate foreground gene sets and their corresponding TRMs have yet to be defined.

What modes of regulation are responsible for targeting these non-C1/PLE FC genes? Certainly, the Ras signal transduction cascade is likely to play a central role, as activation of the Ras/MAPK signaling cascade stimulates generalized FC gene expression [21]. However, only a small fraction of FC genes are up-regulated in a Pnt gof background. We can envision three possibilities for Ras-dependent, but not Pnt-dependent, gene regulation. First, Ras may activate an Ets-domain TF other than Pnt. As the *Drosophila* genome encodes eight total Ets domain proteins, it is possible that one of these Ets TFs is acting downstream of Ras signaling to regulate FC gene expression. However, we observed negligible enrichment for the Pnt (Ets) motif when examining the class of all FC genes. Thus, it seems unlikely that another Ets-domain TF is regulating non-C1 or non-PLE genes. Second, it is possible that the genes of C1 are directly targeted by Pnt, and that the remaining FC genes are genetically downstream of C1. This model places Pnt at the top of the FC gene regulatory hierarchy. However, Pnt lof mutants are lacking only a subset of embryonic muscles, a phenotype inconsistent with this key role of Pnt in all FCs [38,47]. Third, Ras signaling is known to affect multiple transcriptional pathways and either directly or indirectly may activate a non-Ets domain TF to regulate FC gene expression. In fact, there are 20 TFs in the list of 159 FC genes. One or more of these TFs could be target(s) of Ras/MAPK signaling and regulate FC gene expression in the non-Pnt-responsive FCs.

We are now working to expand upon the initial analysis presented here both computationally and experimentally in order to refine our determination of *cis* regulatory codes specific for FC gene expression. First, the genome sequences of many additional *Drosophila* species will be available in the near future [48], and our computational tool for evaluating binding site clustering and evolutionary conservation (ModuleFinder) will need to be extended to incorporate these additional genomes. This can be done most carefully through the use of tree-based methods as suggested by Moses et al. [49], and we are utilizing aspects of their approach in the development of a newer, improved computational tool for quantifying binding site clustering and conservation (Warner et al., manuscript in preparation). ModuleFinder was important for the current work only as a method of quantifying binding site enrichment, and an approach that better utilizes evolutionary conservation can be expected to sharpen the results presented here. Second, in order to resolve the *cis*-regulatory codes driving sub-domains of expression within FCs, two additional data sets are required—higher resolution expression maps for single FC genes, and the DNA binding specificities of additional TFs known to be expressed in FCs. Of the 20 known TFs among the list of 159 confirmed FC genes, most have unknown DNA binding specificities which could be determined using protein binding microarrays [50]. This represents an ideal opportunity to expand upon the analyses presented here and even perhaps find *cis*-regulatory codes for non-C1/PLE FC genes. Also, it may allow us to determine more subtle effects regarding which TFs target which subsets of FC genes. For example, the enrichment observed in C1 for the Tin motif might actually not be for Tin itself, but rather for one of the other NK homeodomain family members, *slouch*, *ladybird early,* and *lbl* [51,52], that are known FC genes.

Thus, the work presented here provides a first step toward determining the mechanisms underlying the regulation of gene expression in FCs. Since the formation of the somatic mesoderm is a complex developmental process requiring input from many signal transduction cascades and tissue-specific TFs, it is an ideal model system for developing an integrated experimental and computational framework that can be applied more generally to identifying *cis-*regulatory codes in animal genomes.

## Materials and Methods

### Promoter analysis.

We obtained 1-kb regions flanking the transcriptional start sites of each of the 159 FC genes from the University of California Santa Cruz (UCSC) Genome Browser dm2 assembly (http://genome.ucsc.edu), extending from 800 bp upstream of transcriptional start to 200 bp downstream of transcriptional start (in a second application, we repeated this analysis with 2-kb regions extending from 1800 bp upstream of transcriptional start to 200 bp downstream of transcriptional start). In cases where more than one transcriptional start site was listed, we used the one closest to translational start. We also extracted a corresponding set of 1,590 non-overlapping regions from promoters of non-FC genes. Both foreground and background proximal promoter regions were repeat masked using the repeat masking provided by UCSC genome browser (http://genome.ucsc.edu). Enrichment in the promoters was measured for each motif using the group specificity score of Hughes et al. [27]. For each of the five motifs dTCF, Mad, Pnt, Twi, Tin, we inspected four different versions of the motif: the collection of matches to known binding sites (see Protocol S1), as well as all words matching within 0.5, 1.0, and 1.5 standard deviations of the motif position weight matrix average [27]. No version of any of these motifs was statistically significant using a confidence level of *p* < 0.05, after applying a Bonferroni correction for multiple hypothesis testing.

### Genome pre-processing for all ModuleFinder scans.

As in the promoter analysis, the D. melanogaster genome was obtained from the UCSC Genome Browser dm2 assembly (http://genome.ucsc.edu). All repetitive regions were masked using the repeat masking provided by UCSC; all exons (as determined by the UCSC refGene annotation) were also masked. For all ModuleFinder scans, we utilized the alignments to D. pseudoobscura (dp2) and D. virilis (droVir1), as provided by the UCSC Genome Browser Multiz alignment of 8 genomes (dm2, droYak1, droAna1, dp2, droMoj1, droVir1, apiMel1, anoGam1).

D. melanogaster translational Start and Stop sites were obtained from the UCSC refGene flat files. Because these files contain redundant references to the same gene, all overlapping reading frames were clustered together; the translational Start and Stop of each such clustered gene was defined to be the most distal (i.e., inclusive) of all translational Starts/Stops in that gene cluster. After clustering genes, we defined the “intergenic regions” to be those sequences contained between adjacent gene clusters, and “intronic regions” to be those sequences contained between the translational Start and Stop of gene clusters. We utilize the terms “gene cluster,” “intergenic region,” and “intronic region” throughout this section.

For all ModuleFinder scans, we utilized windows ranging between 700 and 300 bp (increment size of 50 bp), and the dp2 and droVir1 alignments. We used a “wiggle room” of 5 bp for considering binding sites as conserved. For the input dTCF and Pnt binding site motifs, we utilized the set of words matching within one standard deviation of the average position weight matrix score of the known binding sites [27]; for the Mad, Twi and Tin motifs we utilized only the collection of known binding sites (this was done because the known binding sites for Twi and Tin do not have much variability, and so it was not necessary to extrapolate them. Mad is an extremely degenerate motif, and we found that using a cutoff similar to that of dTCF and Ets caused too large a fraction of sequence space to be considered a motif).

### Analysis of flanking gene length and generation of a length-matched background sequence set.

For each FC gene we matched it to its corresponding gene cluster. Here, although the original list of FC genes from Estrada et al. contained 160 genes, our list contains only 159 since two genes *(CG6682* and *CG13789)* mapped to the same gene cluster. For each gene cluster we then computed the amount of non-coding, non-repetitive sequence in the two intergenic and intronic regions associated with it; thus, each intergenic region is assigned to the two gene clusters that flank it. Detection rates for the 159 FC genes, as well as all other genes are shown in Figure 2A (see next section for details of how the “detection rate” curves are generated).

Because we observed that the 159 FC genes in general had more non-coding, non-repetitive sequence associated with them than other D. melanogaster genes, we generated a length-matched set of background sequences. Here, we wanted to make sure that the distribution of sequence lengths between the foreground and background sets were nearly identical. Since in all following analyses we utilize statistics based on rank-orderings of the foreground and the background gene sets, we sought to make the foreground gene set as uniformly distributed as possible with respect to the background gene set when ranking genes by the length of their associated non-coding, non-repetitive sequences. For this, we first partitioned the D. melanogaster genome into intergenic and intronic regions. We then ordered the regions in each set by length. For the intergenic set of regions, we defined the “foreground regions” to be those regions upstream or downstream of one of the 159 FC genes, and we defined the “non-foreground regions” to be the collection of all other regions (i.e., intergenic regions not upstream or downstream of an FC gene). For each foreground region, we took the seven non-foreground regions occurring directly above and below it in the length-based ranking as background regions (we found that seven was the largest number that could be used and still produce a well-matched background set). In the event that two or more foreground regions did not have 14 background regions ranked between them, we continued to extend above and below them so that the center of this local collection of background regions was the same as the center of their associated foreground regions. Hence, for each foreground region, we were able to associate to it exactly 14 length-matched background regions. We then repeated this matching for the intronic regions to obtain a collection of 14 length-matched background intronic regions for each foreground intronic region. Finally, we concatenated the two intergenic and intronic regions of each foreground gene, as well as the two background intergenic and intronic regions associated with it (note that the background regions that are concatenated need not be adjacent to each other in the D. melanogaster genome, but for simplicity we shall still refer to them as “background genes”). In Figure 2B, the detection rate curves for the concatenated foreground and background genes are shown, and it can be seen that the distributions of their lengths are well matched. Note that for the subsequent analyses involving subsets of the 159 FC genes, we utilized subsets of this background set. Thus, any subset of the 159 FC genes is compared to its associated length-matched subset of background genes, which always contains 14 times as many sequences as the foreground.

### Detection rate curves and relation to WMW.

In Figures 2–6, detection rate curves are shown. In each, foreground and background genes are first pooled together and ordered by a continuous variable (either ModuleFinder score, length, or change in gene expression). Let *F* (respectively, *B*) denote the foreground (respectively, background) set, and let |*F*| (respectively, |*B*|) denote the number of genes in the set. For each *i*∈[1, |*F*|+|*B*|], let δ*_F_*(*i*) be the indicator function that takes the value “1” if the *i*'th-ranked gene is in *F* and “0” otherwise; similarly, let δ*_B_*(*i*) be the corresponding indicator function for *B.* The foreground and background detection rate curves are then defined by


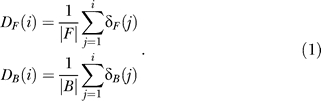


After scaling the X-axis to have a width of 1, the area between the detection rate curves is given by

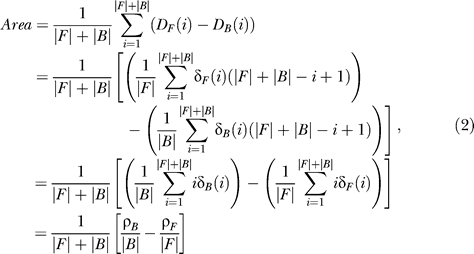
where ρ_F_ and ρ_B_ indicate the sum of the foreground and background ranks. Since

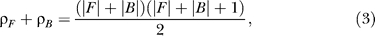
it can be shown that


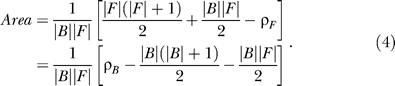


Recall that the WMW test statistic is computed according to the formula [35]

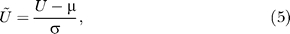
where

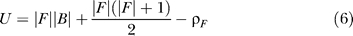
and

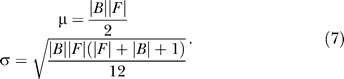
(We note that there is a slightly more complicated formula for σ in the event that there are ties in the rank ordering [35]; we actually utilize this version of σ that corrects for ties in rankings for all computations presented in this paper.) The variable *Ũ* is approximately an *N*(0,1) random variable if the foreground and background sizes are large, and statistical significance is computed by looking at the number of standard deviations into the tail of this normal random variable the test statistic falls. Therefore,


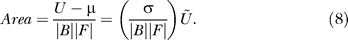


Thus, the area between the detection rate curves is simply a graphical representation of the WMW test statistic, scaled so that only effect sizes (but not sample sizes) are evaluated. Also, note that this measurement is closely related to the area under a receiver-operater curve [53].

Finally, we note that in displaying the detection rate curves, if there are multiple genes with the same score (this frequently occurs, for example, in considering AND combinations where many genes get a score of “0”), then these genes are randomly ordered when plotted. When computing the WMW *p*-value for this enrichment (or in the corresponding area calculation), however, the correction factor for ties is utilized [35].

### Clustering of gene expression microarray data.

All clustering was performed using Cluster [40], and visualized with Java Treeview [54]. We first performed self-organizing map clustering using (Xdim = 1, Ydim = 13, iterations = 100,000) for genes, and (Xdim = 1, Ydim = 4, iterations = 20,000) for arrays. We median-centered the columns of this output and hierarchically clustered genes and arrays using a similarity metric of “correlation (uncentered),” and “average linkage.”

### Proneural cluster analysis.

We obtained all genes from the Supplementary Data of Reeves and Posakony [17]. We note that their original list of non-SOP contains 22 (rather than 18) genes, eight of which are pairs of genes occurring adjacent to one another in the genome. These pairs are *(CG3396:CG3096), (CG8328:CG8333), (CG8361:CG8365)* and *(CG3796:CG3827)*. In order to avoid the problem that a ModuleFinder hit occurring between two of these genes could be counted twice, we dropped one element of each pair *(CG3096, CG8333, CG8365, CG3827)*.

For the Ac/Sc motif, we used RCAGSTGN, as stated by Reeves and Posakony [17] (note that the final degenerate position was added at the end in order to prevent the motif from being able to overlap itself). Su(H) binding sites are listed in Protocol S1; for all scans with it, we utilized the set of words matching within 1 standard deviation of the motif average [27]. We note that there are additional known Su(H) binding sites from the *enhancer of split* complex, but these were not utilized to avoid circularity, as many genes from this complex are PNC genes.

### Validation of predicted enhancers.

CRM regions were PCR-amplified in duplicate, sequence verified, subcloned into the pETW-nuclacZ reporter vector and then injected into *y w* embryos as previously described [19]. At least four independent insertion lines were assessed for each reporter construct. Immunohistochemistry, in situ hybridization, and fluorescent in situ hybridization followed standard protocols [55].

## Supporting Information

Figure S1 Detection Rate Curves Using as a Foreground Gene Set 152 Genes That Are FC but Not PNC Genes Foreground and background regions were searched using the motif combination Su(H) OR Ac/Sc.
(1.0 MB DOC)

Protocol S1 Supplementary Methods Binding sites and sources for dTCF, Mad, Ets, Twist and Tin, SuH
(235 KB DOC)

Table S1 Areas for All Boolean Combinations of the Motifs dTCF/Mad/Pnt/Twi/Tin Using as Foreground Gene Sets C1, PLE, and PTE (25 KB XLS)

Table S2 Information on All Tested Candidate CRMs (51 KB XLS)

## Abbreviations

Ac/Sc: Achaete/Scute
Arm: *armadillo*
Arm+Ras: *armadillo* and *Ras* gof
C1: Cluster 1
CRMs: cis regulatory modules
Dl: *Delta*
Dpp: Decapentaplegic
dTCF: T cell factor
EGFR: epidermal growth factor receptor
*eve*: even-skipped
FC: founder cell
FGFR: fibroblast growth factor receptor
gof: gain-of-function
*hbr*: heartbroken
*lbl*: ladybird late
Lmd: *Lameduck*
lof: loss-of-function
Mad: Mothers against dpp
*mib2*: mindbomb2
N: Notch
*Ndg*: Nidogen
*phyl*: phyllopod
PNCs: proneural cluster
Pnt: Pointed
PTE: Pnt trailing edge
SOPs: sensory organ precursors
Spi: *spitz* lof
Su(H): Suppressor of Hairless
TF: transcription factor
TRM: transcriptional regulatory model
TFBS: transcription factor binding site
Tin: Tinman
Tkv: *thickveins* gof
Twi: Twist
UCSC: University of California Santa Cruz
Wg: Wingless
WMW: Wilcoxon-Mann-Whitney
*vg*: vestigial
VM: visceral mesoderm
