# Supplementary material for: Expression-Guided In Silico Evaluation of Candidate Cis Regulatory Codes for Drosophila Muscle Founder Cells
Source: PLoS Comput Biol. 2006 May 26;2(5):e53. doi: 10.1371/journal.pcbi.0020053 (PMC1464814; doi:10.1371/journal.pcbi.0020053)
Supplement: Figure S1 — Foreground and background regions were searched using the motif combination Su(H) OR Ac/Sc. (1.0 MB DOC) [file pcbi.0020053.sg001.doc]

**Supplementary Figure S1:**

**Supplementary Figure 1:** Detection rate curves using as a foreground gene set 152 genes that are FC but not PNC genes. Foreground and background regions were searched using the motif combination Su(H) OR Ac/Sc.
