# Supplementary material for: Expression-Guided In Silico Evaluation of Candidate Cis Regulatory Codes for Drosophila Muscle Founder Cells
Source: PLoS Comput Biol. 2006 May 26;2(5):e53. doi: 10.1371/journal.pcbi.0020053 (PMC1464814; doi:10.1371/journal.pcbi.0020053)
Supplement: Protocol S1 — Binding sites and sources for dTCF, Mad, Ets, Twist and Tin, SuH (235 KB DOC) [file pcbi.0020053.sd001.doc]

# Supplementary Methods

**Binding sites and sources for dTCF, Mad, Ets, Twist and Tin, SuH**

**dTCF**

CTTTGATCT [1]

GTTTGATGT [1]

CTTTGATCT [2]

CTTTGATAT [2]

CTTTGATCT [3]

GTTTGATCC [4]

CTTTGAAGT [4]

GTTTGATAT [4]

CTTTGATTG [4]

CTTTGAAAA [4]

CTTTGATCT [5]

CTTTGGATG [5]

CTTTAATGG [5]

**Mad**

GCCCCGTC [6]

GACGAGAC [6]

GCCGCCGA [6]

GTCGCCGC [6]

GCCGGTCT [6]

GCCCTGGA [6]

GCCGCTGT [6]

GCCTCCCA [6]

GGCCGGCA [7]

GCCGTCGC [8]

GCCGGCGC [8]

GCCGCCGT [8]

GACGCCAG [8]

GCCGTGGG [8]

GGCGTCAG [9]

GCCGCAGA [3]

GCCGAACC [3]

GCAGCCGC [3]

GCCGCAGC [3]

GTCGTCGC [3]

**Ets**

CAGGAAAC [10]

TGGGATGT [10]

CAGGATAT [10]

CCGGAAAA [10]

CCGGATAT [11]

GGGGAAGC [11]

GAGGAAGT [11]

GTGGATGC [11]

CCGGATGC [11]

CGGGAAAC [12]

CCGGATGC [3]

CCGGAAGC [3]

ATGGATGC [3]

CCGGATCT [3]

**Twist**

CATATG [13]

CATGTG [14]

CATATG [14]

CATGTG [15]

CATCTG [3]

CATATG [3]

**Tin**

CACTTGA [13]

CACTTGA [16]

CACTTGA [16]

CAATTAA [3]

CACTTCA [3]

CACTTAA [3]

CACTTGG [3]

**SuH:**

CGTGGGAC [17]

AATGGGAA [17]

AGTGTGAA [17]

TGTGGGAA [17]

CGTGGGAA [17]

GATGGGAA [17]

CGTGAGAA [17]

TGTGAGAA [17]

TGTGGGAA [17]

GGTGGGAA [17]

TGTGGGAA [18]

CGTGTGAA [18]

CGTGGGAA [18]

TGTGAGAA [18]

TGTGAGAA [18]

CGTGTGAA [18]

CGTGGGAA [18]

CGTGGGAG [18]

CGTGAGAA [19]

TGTGGGAT [10]

AGTGAGAA [10]

TGTGAATA [10]

TATGGGAA [10]

CGTGAGTA [10]

# List of 159 *in situ*-validated FC genes

| CG17492  CG32096  CG12908  CG3758  CG14207  CG13425  CG3365  CG7223  CG10275  CG7867  CG10108  CG1921  CG10250  CG6531  CG11202  CG12052  CG3340  CG33103  CG8147  CG3839  CG31043  CG31317  CG4722  CG6113  CG18250  CG9786  CG3956  CG17724  CG6234  CG4444  CG7958  CG3779 | CG1225  CG3886  CG17871  CG31794  CG10197  CG31151  CG5295  CG4937  CG3166  CG3653  CG14622  CG17342  CG1447  CG9520  CG3619  CG3048  CG7004  CG2328  CG4944  CG32560  CG6534  CG8581  CG8580  CG5522  CG6520  CG15319  CG7250  CG8780  CG7892  CG17952  CG9461  CG7471 | CG4029  CG9952  CG10619  CG11100  CG11280  CG9398  CG4125  CG4531  CG3780  CG3036  CG6464  CG3385  CG6995  CG7392  CG10545  CG17943  CG3936  CG9704  CG2083  CG8597  CG18402  CG12249  CG33529  CG31640  CG8384  CG7852  CG16738  CG4609  CG8166  CG8715  CG30040  CG12306 | CG12530  CG2865  CG30122  CG1911  CG9135  CG4548  CG1311  CG9139  CG7230  CG14447  CG4747  CG9797  CG17556  CG11312  CG6725  CG31536  CG5405  CG7109  CG7752  CG2218  CG10967  CG5069  CG2086  CG1825  CG7187  CG8427  CG12269  CG3606  CG8651  CG7269  CG9554  CG9191 | CG2512  CG10364  CG5408  CG10522  CG8333  CG3938  CG11397  CG17383  CG3800  CG32542  CG18740  CG2890  CG5235  CG3258  CG17046  CG32434  CG9985  CG30115  CG4707  CG3830  CG5723  CG31369  CG1849  CG10388  CG6454  CG17278  CG6570  CG8376  CG5080  CG6545  CG1897 |
| --- | --- | --- | --- | --- |

**FC genes from the Pnt leading edge (PL**E)

| CG10108  CG10250  CG10275  CG12908  CG14207 | CG14447  CG17278  CG17492  CG17556  CG18250 | CG1921  CG2328  CG3166  CG33103  CG3365 | CG3839  CG4937  CG4944  CG6113  CG6520 | CG6531  CG7867  CG8147  CG9139  CG9520 |
| --- | --- | --- | --- | --- |

**FC genes from the Pnt trailing edge (PT**E)

| CG2865  CG31794  CG2086  CG3780  CG17724  CG3258  CG8376  CG8580  CG13425  CG4548  CG4029  CG8651  CG10388  CG3938  CG33529  CG7892 | CG8581  CG9461  CG18402  CG6534  CG11312  CG6454  CG8384  CG6725  CG11202  CG8597  CG31317  CG31640  CG32434  CG7269  CG9797  CG6545 | CG7392  CG1447  CG6995  CG1911  CG7230  CG18740  CG31536  CG9952  CG9786  CG3619  CG1825  CG17383  CG9135  CG3936  CG7187  CG3956 | CG11397  CG3779  CG12530  CG10545  CG5522  CG7250  CG17342  CG10522  CG2890  CG7109  CG2512  CG7471  CG30122  CG11280  CG1311  CG7752 | CG3036  CG3048  CG4722  CG3758  CG17943  CG1897  CG12306  CG9191  CG3800  CG3606  CG8427  CG4747  CG16738  CG10364  CG4609  CG9704 |
| --- | --- | --- | --- | --- |

**FC genes from C1**

| CG33529  CG10197  CG3340  CG3758  CG32096  CG1921  CG1225  CG3839 | CG6531  CG3365  CG17492  CG12908  CG10108  CG14207  CG10250  CG7223 | CG4937  CG6113  CG3653  CG17952  CG31151  CG18250  CG7867  CG10275 | CG11202  CG17871  CG4944  CG3166  CG5295  CG8581  CG7004  CG7892 | CG2328  CG7958  CG33103  CG15319  CG9461 |
| --- | --- | --- | --- | --- |

**References:**

1. Riese J, Yu X, Munnerlyn A, Eresh S, Hsu SC, et al. (1997) LEF-1, a nuclear factor coordinating signaling inputs from wingless and decapentaplegic. Cell 88: 777-787.

2. Yang X, van Beest M, Clevers H, Jones T, Hursh DA, et al. (2000) decapentaplegic is a direct target of dTcf repression in the Drosophila visceral mesoderm. Development 127: 3695-3702.

3. Halfon MS, Carmena A, Gisselbrecht S, Sackerson CM, Jimenez F, et al. (2000) Ras pathway specificity is determined by the integration of multiple signal-activated and tissue-restricted transcription factors. Cell 103: 63-74.

4. Lee HH, Frasch M (2000) Wingless effects mesoderm patterning and ectoderm segmentation events via induction of its downstream target sloppy paired. Development 127: 5497-5508.

5. Knirr S, Frasch M (2001) Molecular integration of inductive and mesoderm-intrinsic inputs governs even-skipped enhancer activity in a subset of pericardial and dorsal muscle progenitors. Dev Biol 238: 13-26.

6. Xu X, Yin Z, Hudson JB, Ferguson EL, Frasch M (1998) Smad proteins act in combination with synergistic and antagonistic regulators to target Dpp responses to the Drosophila mesoderm. Genes Dev 12: 2354-2370.

7. Certel K, Hudson A, Carroll SB, Johnson WA (2000) Restricted patterning of vestigial expression in Drosophila wing imaginal discs requires synergistic activation by both Mad and the drifter POU domain transcription factor. Development 127: 3173-3183.

8. Kim J, Johnson K, Chen HJ, Carroll S, Laughon A (1997) Drosophila Mad binds to DNA and directly mediates activation of vestigial by Decapentaplegic. Nature 388: 304-308.

9. Szuts D, Eresh S, Bienz M (1998) Functional intertwining of Dpp and EGFR signaling during Drosophila endoderm induction. Genes Dev 12: 2022-2035.

10. Flores GV, Duan H, Yan H, Nagaraj R, Fu W, et al. (2000) Combinatorial signaling in the specification of unique cell fates. Cell 103: 75-85.

11. Xu C, Kauffmann RC, Zhang J, Kladny S, Carthew RW (2000) Overlapping activators and repressors delimit transcriptional response to receptor tyrosine kinase signals in the Drosophila eye. Cell 103: 87-97.

12. Rohrbaugh M, Ramos E, Nguyen D, Price M, Wen Y, et al. (2002) Notch activation of yan expression is antagonized by RTK/pointed signaling in the Drosophila eye. Curr Biol 12: 576-581.

13. Lee YM, Park T, Schulz RA, Kim Y (1997) Twist-mediated activation of the NK-4 homeobox gene in the visceral mesoderm of Drosophila requires two distinct clusters of E-box regulatory elements. J Biol Chem 272: 17531-17541.

14. Yin Z, Xu XL, Frasch M (1997) Regulation of the twist target gene tinman by modular cis-regulatory elements during early mesoderm development. Development 124: 4971-4982.

15. Cripps RM, Black BL, Zhao B, Lien CL, Schulz RA, et al. (1998) The myogenic regulatory gene Mef2 is a direct target for transcriptional activation by Twist during Drosophila myogenesis. Genes Dev 12: 422-434.

16. Gajewski K, Kim Y, Lee YM, Olson EN, Schulz RA (1997) D-mef2 is a target for Tinman activation during Drosophila heart development. Embo J 16: 515-522.

17. Morel V, Schweisguth F (2000) Repression by suppressor of hairless and activation by Notch are required to define a single row of single-minded expressing cells in the Drosophila embryo. Genes Dev 14: 377-388.

18. Barolo S, Walker RG, Polyanovsky AD, Freschi G, Keil T, et al. (2000) A notch-independent activity of suppressor of hairless is required for normal mechanoreceptor physiology. Cell 103: 957-969.

19. Kim J, Sebring A, Esch JJ, Kraus ME, Vorwerk K, et al. (1996) Integration of positional signals and regulation of wing formation and identity by Drosophila vestigial gene. Nature 382: 133-138.
